# Neuromorphic Computing with Emerging Antiferromagnetic Ordering in Spin–Orbit Torque Devices

**DOI:** 10.1021/acs.nanolett.4c01712

**Published:** 2024-06-13

**Authors:** Durgesh Kumar Ojha, Yu-Hsin Huang, Yu-Lon Lin, Ratnamala Chatterjee, Wen-Yueh Chang, Yuan-Chieh Tseng

**Affiliations:** † International College of Semiconductor Technology, National Yang-Ming Chiao Tung University, Hsinchu 30010, Taiwan, ROC; ‡ Magnetics and Advance Ceramics Lab, Department of Physics, Indian Institute of Technology Delhi, Hauz Khas, New Delhi 110016, India; § Department of Materials Science & Engineering, National Yang-Ming Chiao Tung University, Hsinchu 30010, Taiwan, ROC; ∥ National University of Science and Technology MISiS, Leninskiy Prospect 4, 119991 Moscow, Russia; ⊥ Powerchip Semiconductor Manufacturing Corporation, Hsinchu 30010, Taiwan, ROC; 6 Industry Academia Innovation School, National Yang-Ming Chiao Tung University, Hsinchu 30010, Taiwan, ROC

**Keywords:** spin−orbit torque, exchange bias, neuromorphic computing, antiferromagnetic, NiO

## Abstract

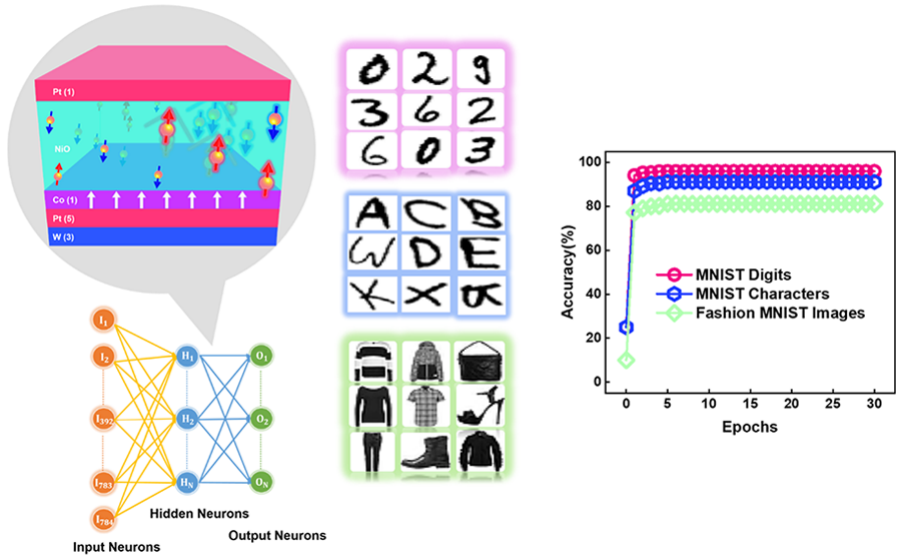

Field-free switching (FFS) and spin–orbit torque (SOT)-based neuromorphic characteristics were realized in a W/Pt/Co/NiO/Pt heterostructure with a perpendicular exchange bias (*H*_EB_) for brain-inspired neuromorphic computing (NC). Experimental results using NiO-based SOT devices guided the development of fully spin-based artificial synapses and sigmoidal neurons for implementation in a three-layer artificial neural network. This system achieved impressive accuracies of 91–96% when applied to the Modified National Institute of Standards and Technology (MNIST) image data set and 78.85–81.25% when applied to Fashion MNIST images, due presumably to the emergence of robust NiO antiferromagnetic (AFM) ordering. The emergence of AFM ordering favored the FFS with an enhanced *H*_EB_, which suppressed the memristivity and reduced the recognition accuracy. This indicates a trade-off between the requirements for solid-state memory and those required for brain-inspired NC devices. Nonetheless, our findings revealed opportunities by which the two technologies could be aligned via controllable exchange coupling.

Magnetization switching based on spin–orbit torque (SOT) is an efficient approach to low-power, high-speed computing and the fabrication of large-scale data storage devices.^1−4^ However, conventional SOT devices for perpendicular magnetization switching typically depend on an external magnetic field to disrupt switching symmetry or minimize the Dzyaloshinskii–Moriya interaction effective field.^5−7^ For SOT-based devices, an external field markedly decreases the storage density, impedes device integration, and limits scalability. In the era of expanded memory density, field-free switching (FFS) is crucial for the practical application of SOT-based devices. Several methods have been devised for the realization of FFS in SOT-based logic devices.^8−25^ One approach involves lateral structural asymmetry, wherein the current-induced effective field is aligned in the out-of-plane direction.^8^ Another method involves the utilization of two heavy metals with contrasting spin Hall angles, which generates competing spin currents to facilitate FFS.^9^ FFS can also be achieved by an in-plane effective field introduced by an electric current and interlayer exchange coupling.^10−12^ Researchers have recently achieved FFS using an out-of-plane (OOP) polarized spin current produced by single-crystal WTe_2_,^13^ the interface between a ferromagnetic (FM) and normal metal,^14,15^ and the interface between an antiferromagnetic (AFM) insulator and heavy metal (HM).^16−19^ Nonetheless, multiple research teams have suggested that the most efficient approach to FFS involves the exchange bias (EB) effect induced by interfacial exchange coupling between FM/AFM layers.^20−25^ Many groups have achieved FFS using EB-based heterostructures, such as Pt/Co/IrMn,^20^ IrMn/CoFeB/MgO,^21^ PtMn/[Co/Ni]_2_/Co/MgO,^22^ Ta/Pt/CoFe/IrMn/Pt,^23^ or NiO/Pt/Co/Pt.^24^ Grochot et al.^25^ recently reported on the substitution of an external field with an in-plane EB field, thereby enabling field-free perpendicular magnetization switching of the Co layer when paired with NiO as an AFM layer in a W(Pt)/Co/NiO system. This approach exhibits current-induced SOT switching of the perpendicular magnetized FM layer as well as gradual switching behavior under a pulsed electric current (i.e., memristive characteristic), which makes it a strong candidate for neuromorphic computing (NC).

By mimicking the functions of the human brain, NC devices can perform cognitive tasks such as recognition and reasoning, in a highly efficient manner. This can be attributed to massive parallel processing and high energy efficiency.^26^ Several reports have suggested that SOT-based devices could be used as artificial synapses for NC.^11,22,27−32^ Yadav et al. experimentally constructed LTD/LTP responses for the Pt/Co/SiO_2_ SOT system and utilized it as an artificial synapse for NC.^33^ Kurenkov et al. realized an artificial neuron and synapse in an AFM/FM-based SOT device for NC.^34^ Zhou et al.^35^ employed a SOT-based bilayer L1_1_-CuPt/CoPt as an artificial sigmoidal neuron in an artificial neural network (ANN) for NC applications. Li et al. demonstrated a sigmoidal neuron using a ferrimagnetic Co/Gd device for NC.^36^ Recently, Cao et al. developed a SOT-based W/CoFeB/MgO device to recognize MNIST data sets under fully constructed artificial synapses and neurons.^37^ Similarly, Dong et al. employed L1_0_ FePt/TiN/NiFe devices in the construction of artificial synapses and sigmoidal neurons for implementation in an ANN for NC.^11^

Unlike magnetic memory technology that emphasizes FFS, a low switching current density, and perpendicular magnetic anisotropy (PMA), an NC device appeals for linearity, plasticity, and memristivity for a high recognition accuracy.^38−42^ In this study, we developed a NiO-based SOT heterostructure (W/Pt/Co/NiO/Pt) with NiO layers of various thicknesses. In these devices, the perpendicular magnetization of the Co (FM) layer was switched via a mechanism that involved current-induced SOT under modified interfacial coupling with varying NiO-AFM orderings. We tracked the evolution of the interfacial coupling in terms of switching properties from the perspectives of memory and NC devices. Samples with a thick NiO layer presented well-developed AFM ordering and coupling via the Co layer in terms of the perpendicular EB (*H*_EB_) effect. Current-induced SOT switching loops and Hall resistance (*R*_Hall_) versus pulse current response counts were used to construct artificial sigmoidal neurons and synapses for implementation in an ANN, respectively. The proposed system was then assessed in terms of image recognition accuracy when applied to MNIST handwritten data sets and Fashion MNIST.

W/Pt/Co/NiO/Pt heterostructures were fabricated on Si/SiO_2_ substrates using an ultrahigh vacuum (UHV) sputtering system at room temperature. The thickness of the various layers was as follows: 3 nm for W, 5 nm for Pt, 1 nm for Co, 2, 15, or 30 nm for NiO, and 1 nm for Pt. The three samples were named according to the thickness of the NiO layer (the independent parameter) as follows: NiO-2 nm, NiO-15 nm, and NiO-30 nm. Details about the experiments and measurement techniques can be found in section S1 of the Supporting Information. The crystallinity of the NiO-based sputtered heterostructures was characterized by performing grazing incidence wide-angle X-ray scattering (GIWAXS) measurements using a two-dimensional (2D) detector.^43−46^ Note that GIWAXS is a conventional scattering method commonly used to investigate the crystal structures of thin films. Using angle-resolved data, this method exposes diffraction signals associated with the surface and grain orientations of the sample, covering a range from in-plane to out-of-plane (OOP) directions of the films. Panels a–c of Figure 1 present GIWAXS patterns of NiO-2 nm, NiO-15 nm, and NiO-30 nm samples, respectively, while panel d illustrates the extracted angle-resolved X-ray diffraction (ARXRD) peaks at a Ψ of 0°. Details about the GIWAXS patterns are described in section S2. The ARXRD patterns of samples with a thin NiO layer (2 nm) correspond to the strong polycrystalline nature of Pt, whereas the samples with a thick NiO layer (15 or 30 nm) correspond to NiO polycrystalline planes.^25,44^ Samples NiO-15 nm and NiO-30 nm presented clear evidence of highly ordered NiO (111) crystallographic planes aligned along the OOP direction (Ψ = 0°). This implies that the crystal orientation tended to align in the perpendicular direction. The importance of this lies in the crystalline characteristics of the (111) plane within the NiO layer, in which the spins of alternating (111) planes are associated with AFM. The AFM crystallographic ordering of sample NiO-30 nm was more robust than that of sample NiO-15 nm. This resulted in stronger exchange coupling within the Co (FM)/NiO (AFM) bilayer, which contributed to robust perpendicular exchange bias (*H*_EB_).

**Figure 1:**
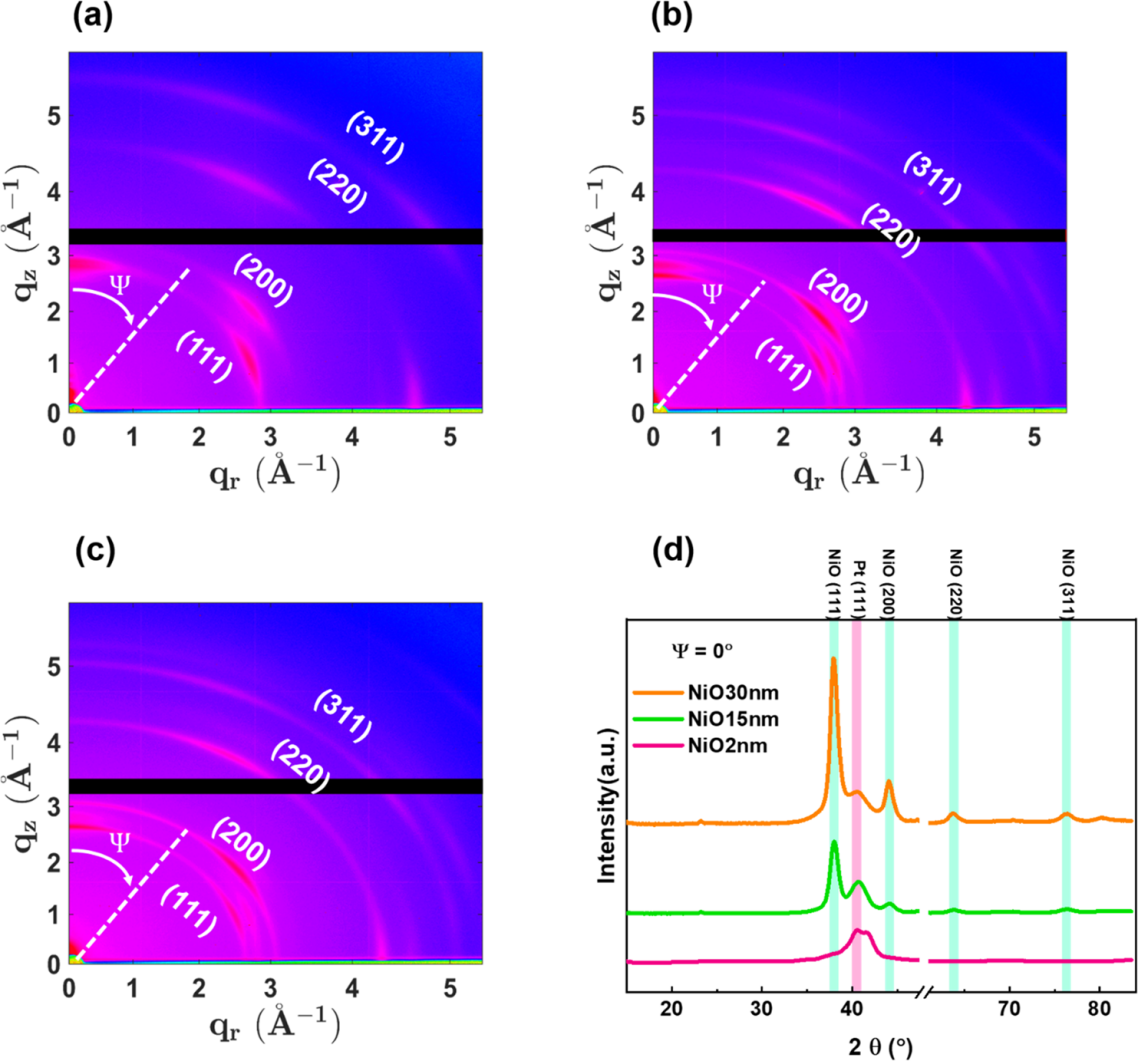
Crystallographic analysis of films with NiO layers of various thicknesses. (a–c) GIWAXS patterns of samples NiO-2 nm, NiO-15 nm, and NiO-30 nm, respectively. All three samples revealed textured Pt and NiO crystallographic planes of (111), (200), (220), and (311). Sample NiO-2 nm presented the crystallographic planes characteristic of Pt, whereas samples NiO-15 nm and NiO-30 nm exhibited the planes of NiO. Ψ indicates the polar angle between the *q*_*z*_ axis and the *q*_r_ plane. In this case, a Ψ of 0° refers to Fourier-integrated diffraction patterns along the out-of-plane direction of the film. (d) 2θ profiles of the three samples derived from their respective GIWAXS patterns. The transformation from the *q* space to the 2θ profile was achieved using a wavelength of 1.5418 Å.

Figure 2a illustrates the setup used to obtain Hall bar-based electrical measurements and the spin configuration of the proposed device. Figure 2b represents the *R*_Hall_ versus perpendicular magnetic field (*H*_*z*_) response of NiO-based devices, and its switching loop confirmed the PMA nature of the Co layer. Panels c–e of Figure 2 present the magnetization hysteresis loop versus *H*_*z*_, which was used to elucidate the EB effect in the NiO-based heterostructures.^47^ For sample NiO-30 nm, the estimated value of *H*_EB_ is ∼90 Oe, while the *H*_EB_ values are negligible for the 2 and 15 nm cases. The absence of *H*_EB_ effects in the NiO-2 nm sample can be attributed to a lack of AFM ordering in the NiO layer. Despite the absence of *H*_EB_ effects in the NiO-15 nm sample, we observed an increase in the coercive field (*H*_c_) indicating weak NiO-AFM, which tended to rotate with the Co-FM layer and thereby expand *H*_c_. Under these conditions, the insignificant effects of *H*_EB_ can be attributed to the coherence of AFM and FM and their rotation as a single layer. In sample NiO-30 nm, the rotation of Co-FM was independent of that of robust NiO-AFM, which decreased *H*_c_ but significantly increased *H*_EB_.

**Figure 2:**
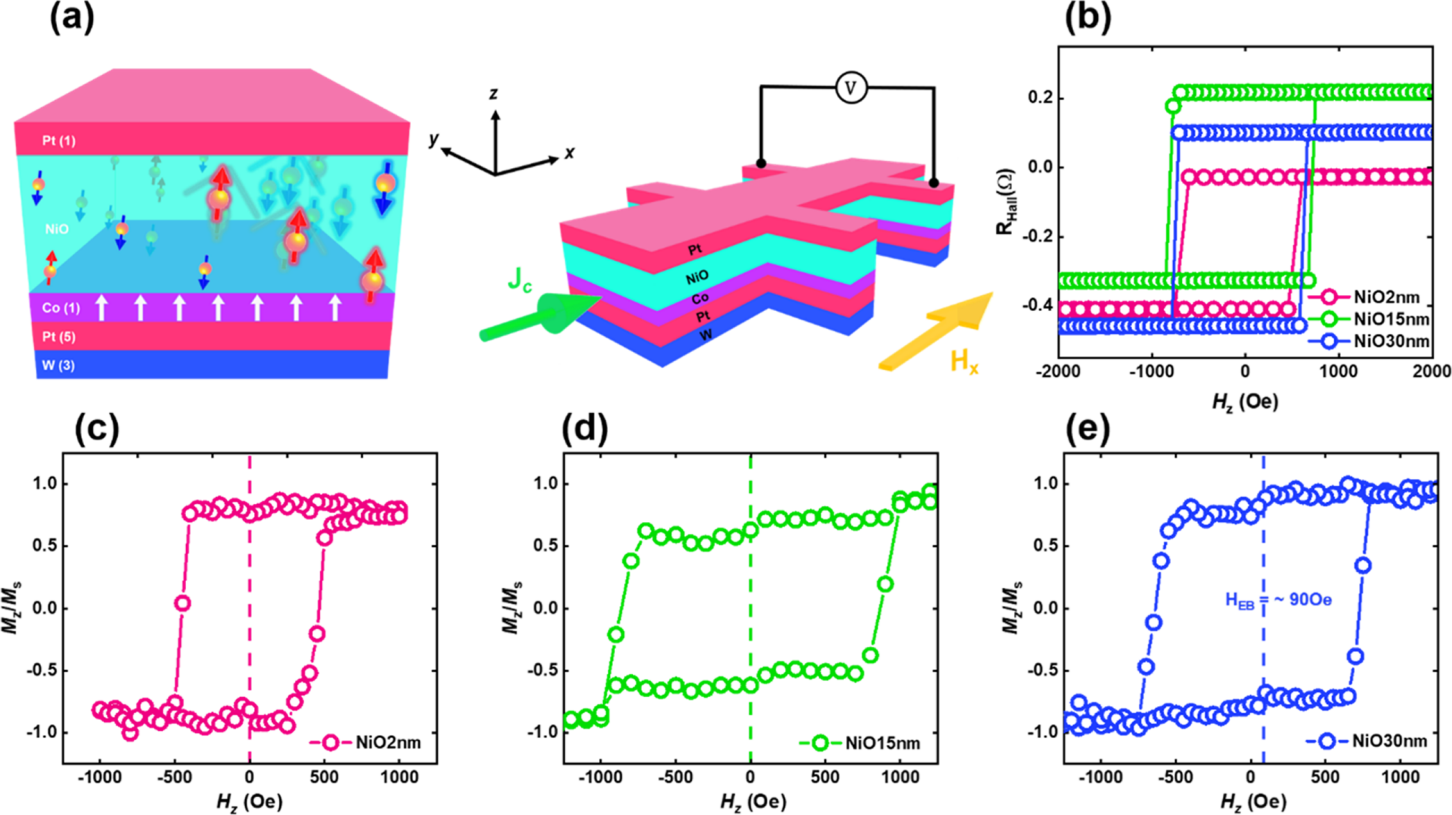
Schematic device structure and perpendicular exchange bias effect. (a) Schematic illustration of the structure of the NiO-based device with the spin configuration (left) and setup for Hall bar electrical measurements (right). (b) Variations in *R*_Hall_ in response to a perpendicular magnetic field (*H*_*z*_), highlighting the PMA nature and switching properties of the NiO structure. (c–e) Magnetic hysteresis loops as a function of *H*_*z*_ revealing perpendicular *H*_EB_ in samples NiO-2 nm, NiO-15 nm, and NiO-30 nm, respectively.

Anomalous Hall effect (AHE) measurements were used to assess the OOP-effective field (*H*_*z*_^eff^) generated by the in-plane current in NiO-based devices (section S3). The *H*_*z*_^eff^ field was computed without the joule heating effect using the expression *H*_*z*_^eff^ = (*H*_c_^+^ + *H*_c_^–^)/2, where *H*_c_^+^ and *H*_c_^–^ are the down-to-up and up-to-down coercive fields, respectively.^7,8,48^ The *H*_*z*_^eff^ value corresponds to the center shift of the AHE loop under positive and negative bias currents. Panels a–c of Figure 3 illustrate the variation of *H*_c_^+^, *H*_c_^–^, and *H*_*z*_^eff^ fields with bias current density (*J*_c_) for the NiO-2 nm, NiO-15 nm, and NiO-30 nm devices, respectively. In all three devices, increasing the bias current decreased the coercivity in the AHE loops, thereby revealing the expected modulation of anisotropy in the Co layer due to the effects of SOT.^48,49^ Samples NiO-2 nm, NiO-15 nm, and NiO-30 nm showed negligible shifts in the *H*_*z*_^eff^ field along the perpendicular (*z*) direction. The difference in *H*_*z*_^eff^ at input currents of −19 and 19 mA for NiO-2 nm is −34 Oe, while it is ∼20 Oe for samples NiO-15 nm and NiO-30 nm at input currents of ±29 and ±15 mA, respectively. The slope of *H*_*z*_^eff^/*J*_c_ under *H*_*x*_ = 0 Oe is insignificant for each NiO-2 nm, NiO-15 nm, and NiO-30 nm device, as illustrated in panels a–c of Figure 3, respectively. This indicates the presence of a small OOP-effective field in our NiO-based SOT device, which cannot facilitate complete FFS without *H*_*x*_.^48,49^ Our results align with those of the previous report.^50^ Sample NiO-2 nm exhibited a weak *H*_*z*_^eff^ field and negligible EB due to a lack of AFM ordering between the thin NiO layer and the Co layer. The insufficient broken inversion symmetry in this configuration hindered field-free-SOT switching. In contrast, the perpendicular EB or AFM ordering in samples NiO-15 nm and NiO-30 nm can facilitate current-induced FFS. This is attributed to either weak AFM ordering (NiO-15 nm) or strong AFM ordering (NiO-30 nm) in the NiO layer. On the basis of this observation, we infer that weak AFM ordering in the NiO-15 nm sample and robust AFM ordering, or a perpendicular *H*_EB_ effect in sample NiO-30 nm, could facilitate FFS.

**Figure 3:**
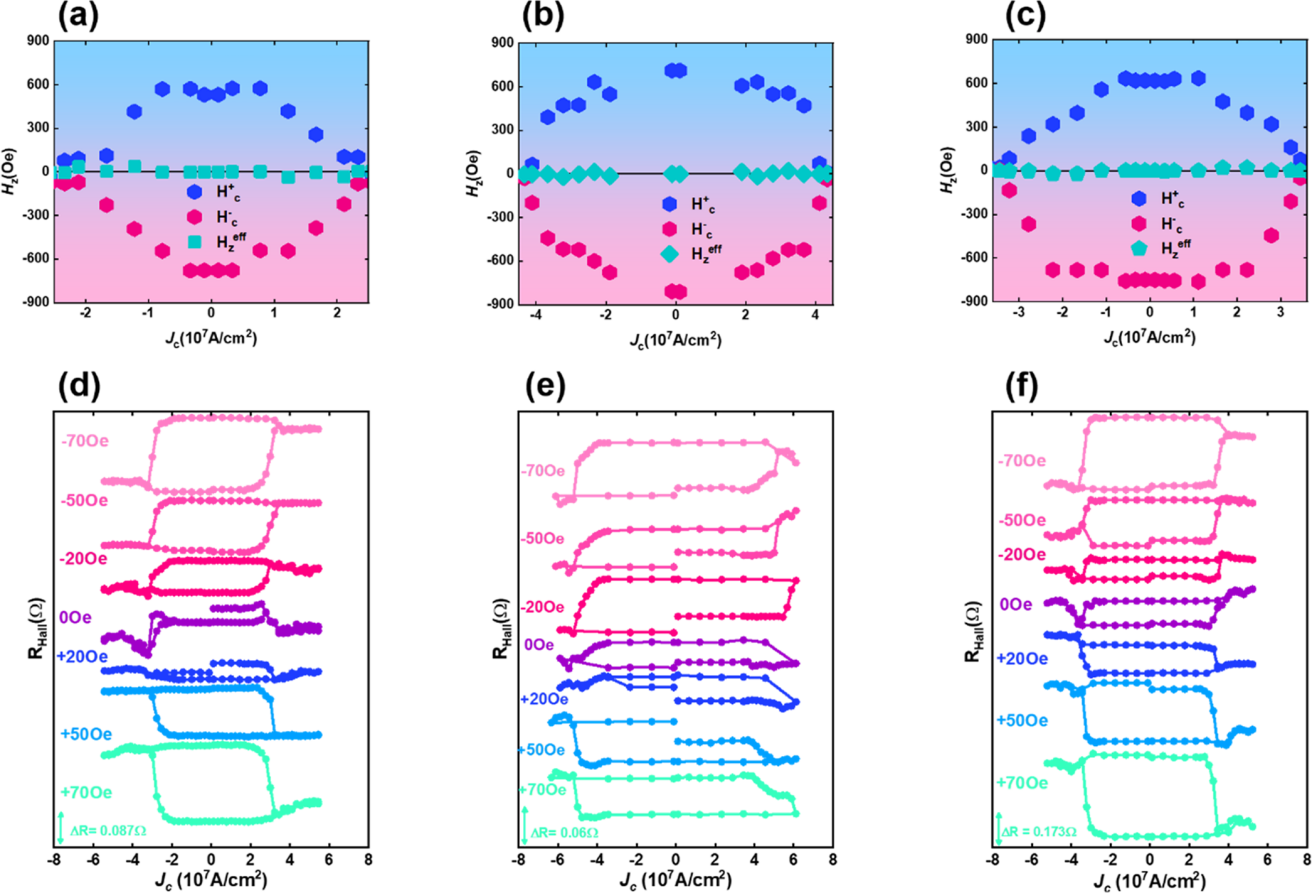
*H*_*z*_^eff^ effect and current-induced magnetization switching loop of NiO-based SOT devices. (a–c) Variations in the switching field of the AHE loop of NiO-2 nm, NiO-15 nm, and NiO-30 nm devices, respectively, under a bias current density. Blue and pink indicate positive (*H*_c_^+^) and negative (*H*_c_^–^) switching fields, respectively, while light green indicates the *H*_*z*_^eff^ of the devices. (d–f) Current-induced SOT switching loop of NiO-2 nm, NiO-15 nm, and NiO-30 nm devices, respectively, with *H*_*x*_ varied from 70 to −70 Oe (bottom to top, respectively). Positive and negative *H*_*x*_ values indicate the clockwise and anticlockwise rotation, respectively, of the switching loop. Purple denotes the switching loop under *H*_*x*_ = 0 Oe for each device. The gradual multiple intermediate states exhibited in this SOT switching loop can be used to construct sigmoidal neurons.

To investigate the impact of the perpendicular *H*_EB_ effect on our proposed perpendicularly magnetized thin Co layer-based NiO devices, we performed measurements of SOT-induced magnetization switching by an electric current, as illustrated in Figure 3d–f.^4,11,23,25^ The achieved SOT switching involved the application of different in-plane bias fields (*H*_*x*_) for symmetry breaking^2,4,51^ (*H*_*x*_ values from −70 to 70 Oe), as illustrated in Figure 3d–f. Panels d–f of Figure 3 illustrate the switching loops of the NiO-based samples as a function of *H*_*x*_. In sample NiO-2 nm, deterministic switching loops were readily noticeable, except where *H*_*x*_ = 0 Oe. This observation is in line with expectations due to the negligible perpendicular *H*_EB_ and *H*_*z*_^eff^ field, which was insufficient to break the switching symmetry. In samples NiO-15 nm and NiO-30 nm, we observed deterministic FFS attributable to the AFM ordering or perpendicular *H*_EB_ effect, as evidenced by ∼8% and ∼10% switching, respectively (section S4). We observed a change in switching polarity from clockwise to counterclockwise under a reversal of *H*_*x*_ from positive to negative, as previously reported in a Pt/Co bilayer system.^4,6^ We also observed gradual switching loops, due to a lack of AFM ordering (in sample NiO-2 nm) or well-developed AFM ordering (in sample NiO-30 nm). By contrast, sample NiO-15 presented obvious switching loops under high current density, including gradual (up-to-down) and nongradual (down-to-up) sharp linear switching loops for both positive and negative *H*_*x*_. This can perhaps be attributed to moderate Co/NiO coupling. Here, the perpendicular *H*_EB_ field corresponding to exchange coupling between the Co and thick NiO layer facilitated FFS by taking on the role of *H*_*x*_. This can be explained by the fact that when *H*_*x*_ = 0 Oe, the initial orientation of Co layer magnetization was toward the −*z* direction. A positive current pulse passing through the Pt layer (along the *x*-axis) induced a spin current with spin polarization, facilitated by the SOT mechanism. The perpendicular EB (directed along the *z*-axis) disrupted mirror symmetry via robust exchange coupling at the Co/NiO interface, which induced magnetization of the Co layer to switch from the down (−*z*) state to the up (*z*) state at a critical current density (*J*_c_). Similarly, when the pulse current was reversed, the Co layer switched from an up state to a down state. In sample NiO-30 nm, the *J*_c_ values for the down-to-up and up-to-down states were 33.9 × 10^6^ and −33.3 × 10^6^ A/cm^2^, respectively. This slight difference in *J*_c_ can be attributed to differences in the barriers, with the discrepancy decreasing under *H*_*x*_. The switching phase diagrams^6^ of all devices are available in section S5.

The perpendicular *H*_EB_ effect in these devices was confirmed by the switching percentage,^23,25^ which reached 65% at ∼500 Oe in sample NiO-30 nm (section S6). Collectively, these findings suggest that the observed gradual (up-to-down) and sharp deterministic FFS in samples NiO-15 nm and NiO-30 nm (section S4) can be attributed to the interplay of weak and strong Co/NiO exchange coupling, respectively. These findings underscore the subtle yet robust AFM ordering of the NiO layer and its intricate connection with the Co layer via exchange coupling.

The current-induced SOT switching loop (Figure 3d–f) exhibits several intermediate magnetic orientations. This multistate switching, or memristive behavior, is crucial for neuromorphic computing.^29,32,34^ We investigated these characteristics by a sweeping pulse current under *H*_*x*_ = −300 Oe, as detailed in section S7. In addition to the switching induced by current-driven SOT, we observed an analog change in the magnetization of the Co layer when the devices were exposed to a pulsed electric current, as shown in Figure 4. This trait could potentially be used as a synapse^11,22,27−32,34,52−55^ for information transmission in high-efficiency NC applications. Figure 4 illustrates the neuromorphic characteristics of NiO-based SOT devices under an applied pulse current. As illustrated in Figure 4a, within the complex framework of the biological brain, synapses serve as pivotal links between neurons. In an ANN, artificial synapses provide crucial connections between neurons, while allowing the adjustment of connection strength via weighting to maintain continuous nonvolatile conductance. The NiO-based device (Figure 4b) is activated by a pulse current sequence along the *x*-axis (Figure 4c). The resulting *R*_Hall_ responses corresponding to NiO-2 nm, NiO-15 nm, and NiO-30 nm under *H*_*x*_ = −300 Oe are presented in panels d–f, respectively, of Figure 4 (section S1). In samples NiO-2 nm and NiO-30 nm, we observed a distinctive pattern of gradual decreases in *R*_Hall_ during consecutive negative current pulses, indicating long-term depression (LTD), and increases during consecutive positive current pulses, indicating long-term potentiation (LTP). In sample NiO-15 nm, LTD and LTP correspond to consecutive negative and positive current pulses, respectively. The mechanism underlying this phenomenon is likely the nucleation and growth of domains within the Hall bar device, as reported previously.^28,56^ Note that in NiO-based devices, the gradual response of *R*_Hall_ versus pulse number (both increasing and decreasing) indicates synaptic behavior that can be applied to NC.^11,34,52−55^

**Figure 4:**
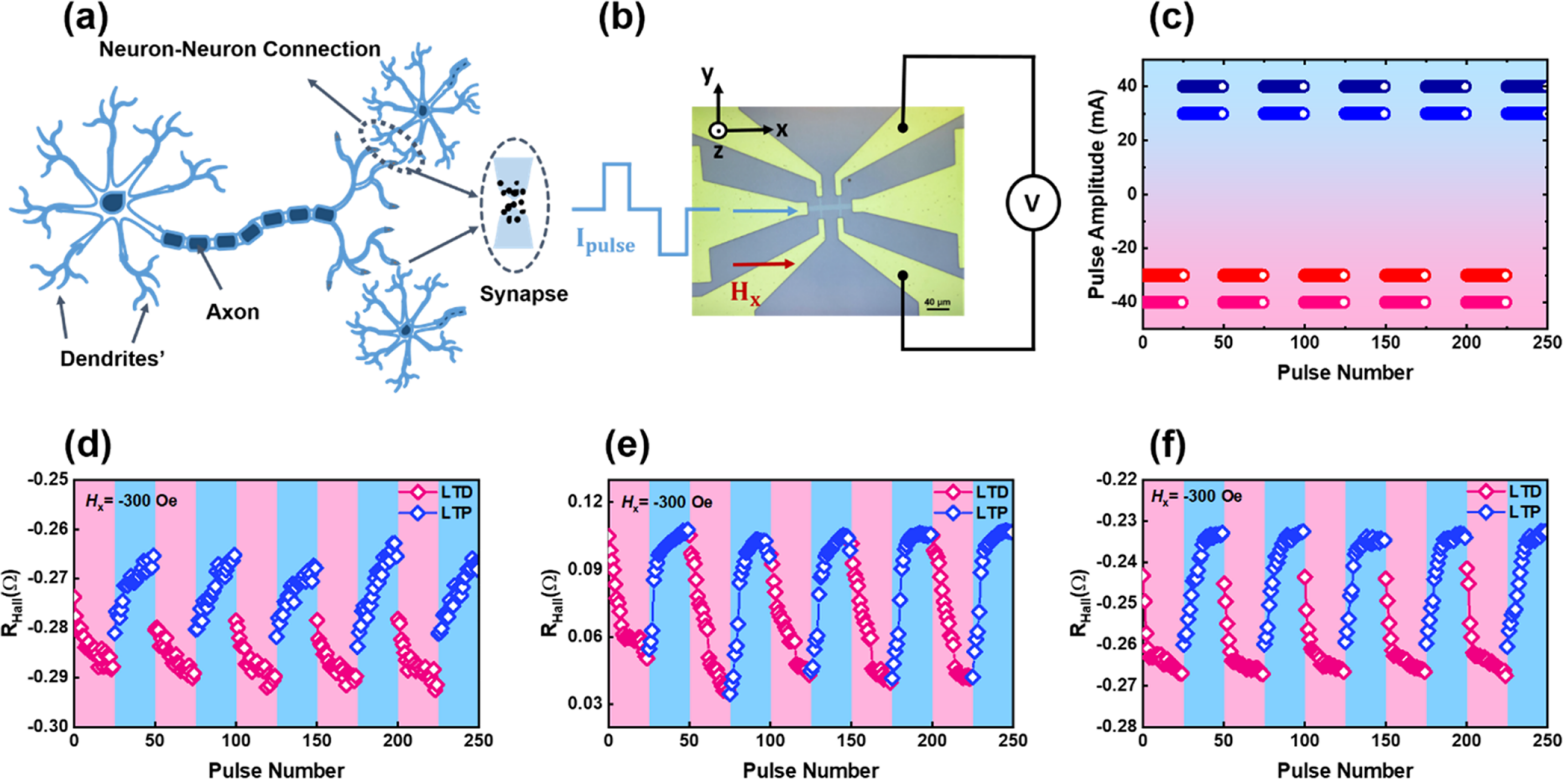
Neuromorphic characteristics of the proposed NiO-based SOT devices under an applied pulse current. (a) Schematic of biological neurons and synaptic connections. (b) Schematic of the fabricated Hall bar device under the setup of electrical measurements. (c) Tuning of the Hall resistance (*R*_Hall_) using a sequence of 25 positive current pulses (blue and dark blue lines) with amplitudes of 40 mA (for NiO-15 nm) and 30 mA (for NiO-2 nm and NiO-30 nm) and a pulse duration of 500 μs, followed by a corresponding set of 25 negative pulses (red and pink lines) with amplitudes of −40 mA (for NiO-15 nm) and −30 mA (for NiO-2 nm and NiO-30 nm) and a pulse duration of 500 μs. For samples NiO-2 nm and NiO-30 nm, we applied consecutive pulse currents of −30 and 30 mA (blue and red lines, respectively), whereas for sample NiO-15 nm, we applied larger pulse currents of −40 and 40 mA (dark blue and pink lines, respectively) due to the large coercivity (*H*_c_). (d–f) *R*_Hall_ response with pulse number (LTD and LTP response) under *H*_*x*_ = −300 Oe for samples NiO-2 nm, NiO-15 nm, and NiO-30 nm, respectively. These experimentally constructed LTD and LTP responses can be used as an artificial synapse for MNIST pattern recognition tasks.

As shown in Figure 5a, we employed current-induced switching measurements under *H*_*x*_ = −300 Oe in the design of artificial neurons featuring a sigmoidal activation function.^11,35^ The process of constructing an analog sigmoid function is outlined in section S8. We observed sharp linear switching loops in the NiO-15 nm device (weak Co/NiO exchange coupling) and NiO-30 nm device (robust Co/NiO exchange coupling), influencing the extraction of the sigmoid function, as shown in Figure 5b. In contrast, the NiO-2 nm device exhibited smooth and gradual switching behavior, due to its thin NiO layer and lack of established Co/NiO exchange coupling, enabling the construction of an optimal sigmoid function. Input current *X* triggers magnetization switching in the Co layer, leading to the conversion of the resulting *R*_Hall_ into output *Y*, as depicted in Figure 5b.^11,35^

**Figure 5:**
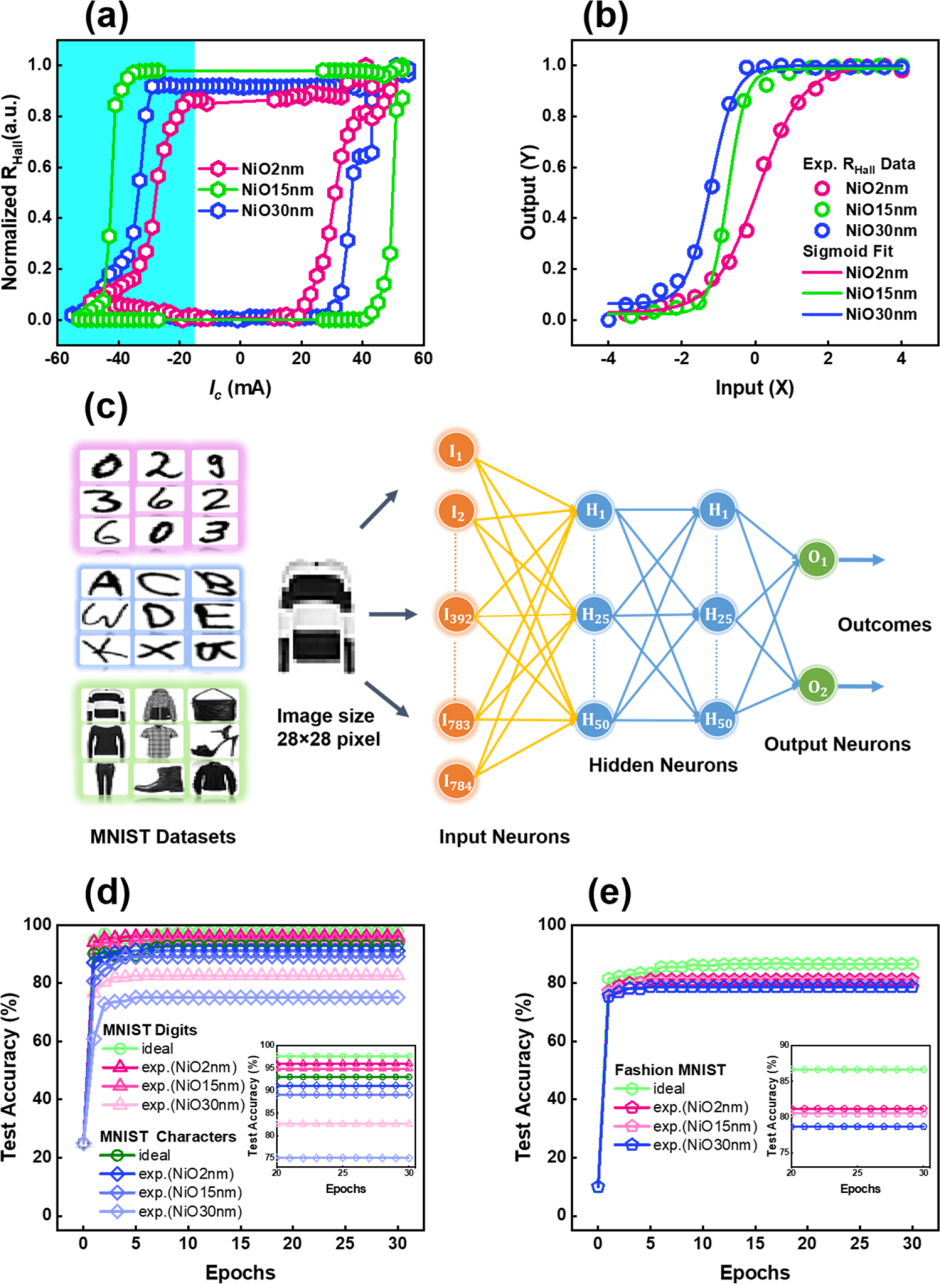
Sigmoidal artificial neuron and ANN assessed in terms of image recognition accuracy when applied to the Fashion MNIST data set. (a) Standard normalized switching loop observed in NiO-based devices under *H*_*x*_ = −300 Oe. The pink, green, and blue circles indicate the switching loop of samples NiO-2 nm, NiO-15 nm, and NiO-30 nm, respectively. The activation function for an artificial neuron in the hidden layer is derived using the sigmoid function from the *R*_Hall_ data points in the shaded region. (b) Optimal *R*_Hall_ data points for the NiO-2 nm, NiO-15 nm, and NiO-30 nm devices were extracted from the shaded region of switching loop measurements under *H*_*x*_ = −300 Oe. These points were used to construct sigmoid functions represented by pink (NiO-2 nm), green (NiO-15 nm), and blue (NiO-30 nm) circles, with their corresponding sigmoid fits depicted by lines of the same colors. (c) ANN for Fashion MNIST image recognition consisting of the input layer (784 neurons), two hidden layers (50 neurons each), and an output layer (two neurons). Accuracy of recognition of (d) MNIST digits and characters and (e) Fashion MNIST data of our NiO-based proposed devices (NiO-2 nm, NiO-15 nm, and NiO-30 nm). The insets of panels d and e show that the NiO-2 nm and NiO-15 nm devices are superior to the NiO-30 nm device in terms of recognition accuracy. The training accuracy for the same MNIST and Fashion MNIST patterns can be found in section S12.

The effectiveness of the proposed devices in NC applications was assessed using experimentally constructed artificial synapses and sigmoidal neurons for implementation in an ANN designed for the recognition of MNIST and Fashion MNIST data set patterns. The multilayer perceptron with gradient descent and backward propagation^11,57^ algorithms was used for training based on a handwritten data set featuring 28 pixel × 28 pixel images of four digits (“1”, “2”, “4”, and “6”) and four characters (“d”, “k”, “y”, and “c”). The network comprised an input layer (784 neurons), two hidden layers (10 neurons each), and an output layer (four neurons) (section S9). The simulation of MNIST pattern recognition accuracy was conducted under two scenarios. The first scenario utilized the ideal sigmoid function as the activation function for the hidden layer with weight updating between the hidden and output layers facilitated by an ideal synapse. Hereafter, this scenario is termed *ideal*. The second scenario was implemented by using an experimentally constructed synapse based on 150 *R*_Hall_ states (Figure 4d–f) for synaptic weight^31,32^ and fitted sigmoidal functions (Figure 5b) as the hidden layer activation function.^11^ The second scenario is experimental and therefore termed *exp*. Each ANN underwent training using 24,460 images of MNIST digits and 19,200 images of MNIST characters. The recognition performance of NiO-based devices was assessed using 4107 test images of digits and 3200 test images of characters (Figure 5d).

In *ideal* case simulations, the ANN achieved high recognition accuracies of 97.66% and 93.06% for MNIST digits and characters, respectively. In *exp* cases, the NiO-2 nm device achieved high recognition accuracies of 96.0% and 91.25% for MNIST digits and characters, respectively. The accuracies of the NiO-15 nm device were slightly lower (94.88% and 89.19%, respectively), whereas the accuracies of the NiO-30 nm device were far lower (82.64% and 75.13%, respectively). The superior accuracy of the NiO-2 nm and NiO-15 nm devices can be attributed to the synaptic behavior (LTD and LTP response) and optimally extracted sigmoid function (section S10).

These results indicate that when using MNIST data, the recognition accuracy depends on a combination of experimentally constructed synapses and sigmoidal neurons, modulated via Co/NiO exchange coupling. This implies that an increasing level of FM–AFM exchange coupling will reduce memristivity (section S7) and thereby undermine NC performance. We also created an ANN with two hidden layers (three neurons each) for recognizing three digits (“2”, “4”, and “6”) and three characters (“d”, “e”, and “p”). With the NiO-2 nm device, we achieved ∼95.4% accuracy for digits and 95.17% for characters (section S11). These results are in line with previous findings.^11,55^ The recognition accuracy of the proposed devices was also assessed using the Fashion MNIST data set.^58^ As shown in Figure 5c, we implemented an ANN utilizing 300 *R*_Hall_ states as synaptic weights under the same sigmoidal neuron. Training was performed using 12 000 images of pullovers and coats, and testing was performed using 2000 corresponding images (section S12). The test accuracy (Figure 5e) varied with the device structure as follows: 81.25% for NiO-2 nm, 80.60% for NiO-15 nm, and 78.85% for NiO-30 nm. Note that these values are close to the ideal test accuracy of 86.75%. Recognition results for MNIST and Fashion MNIST images are given as a function of NiO layer thickness in Table 1. Panels d and e of Figure 5 compare the recognition accuracy of the three devices when applied to the MNIST and Fashion MNIST data sets, respectively.

**Table 1:** Pattern Recognition Train and Test Accuracy of NiO-Based SOT Devices under an Ideal Synapse and Neurons (*ideal* case) and an Experimental Synapse and Sigmoidal Neurons (*exp* case) for MNIST and Fashion MNIST Images

			experimental synapse and neurons			
MNIST data set	type of accuracy	test accuracy of ideal synapses and neurons (%)	NiO-2 nm device test accuracy (%)	NiO-15 nm device test accuracy (%)	NiO-30 nm device test accuracy (%)	synaptic weight
handwritten digits 1, 2, 4, and 6	train	97.60	96.29	94.27	82.96	150*R*_Hall_
	test	97.66	96.00	94.88	82.64	
handwritten characters d, k, y, and c	train	94.18	92.38	90.74	75.80	150*R*_Hall_
	test	93.06	91.25	89.19	75.13	
Fashion MNIST images (sweaters and coats)	train	89.27	83.77	81.20	81.43	300*R*_Hall_
	test	86.75	81.25	80.60	78.85	

Additionally, the four-terminal Hall devices in this study demonstrate the switching behavior of an FM/AFM-based SOT device using Hall signals between distinct spin states, showcasing the scalability potential through advanced lithography techniques. Preserving analog behavior while reducing device dimensions is a primary challenge for spintronic memristors.^27^ Previous research has highlighted the size-dependent nature of SOT switching, emphasizing the impact of domain size.^59^ SOT devices within a few hundred nanometers can preserve gradual domain-based switching, enabling expansive arrays within NC architectures. Addressing scalability issues requires materials engineering for hosting numerous magnetic domains or skyrmions in nanoscale devices. Strategic engineering of domain walls could lead to ultra-high-density neuromorphic computing chips, underscoring the transformative potential of our work in advancing spintronics and NC.

This paper reports on field-free switching and SOT-based neuromorphic characteristics in a W/Pt/Co/NiO/Pt heterostructure with perpendicular *H*_EB_ for brain-inspired neuromorphic computing. AFM–FM exchange coupling was modified via NiO ordering to assess SOT switching and the corresponding NC properties. In the thickest sample (NiO-30 nm), deterministic FFS was achieved under minimal *J*_c_ values of 33.9 × 10^6^ A/cm^2^ in the presence of a perpendicular *H*_EB_ effect. An ANN constructed using artificial synapses and sigmoidal neurons achieved remarkable recognition accuracy when applied to MNIST digits, characters, and Fashion MNIST images. Weak AFM resulted in memristor-like gradual switching, which enhanced NC recognition accuracy due to coherent AFM–FM reversal across the interface. Robust AFM resulted in a more efficient field-free reversal with the assistance of *H*_EB_; however, this was shown to suppress memristivity in terms of multiple *R*_Hall_ states, thereby reducing the NC recognition accuracy. Table 2 compares our case with various SOT-based devices in terms of *J*_c_ values and recognition accuracy when applied to the MNIST and Fashion MNIST data sets. Our findings demonstrate the potential of using SOT-based devices with facile control over interfacial properties in NC devices.

**Table 2:** Benchmarks of MNIST and Fashion MNIST Image Pattern Recognition Accuracy, Critical Current Density, and Types of Experimental Synaptic Weights, Used in Artificial Neural Networks across Various SOT-Based Devices

SOT-based neuromorphic device	FFS switching through	critical current density (×10^7^ A/cm^2^)	experimental synaptic weight used in an ANN	MNIST and Fashion MNIST test accuracy (%)	ref
Ta/GdFeCo/Ta	NA	1.26	*R*_Hall_ vs pulse number	92.25 (four characters)	(52)
Ta/Pt/Co/SiO_2_	NA	3.4	*R*_Hall_ vs pulse number	82.8 (MNIST)	(33)
				71.7 (Fashion)	
[CoTb]Pt/Si_3_N_4_	composition gradient	∼5–6	*R*_Hall_ vs pulse number	94.04 (three characters)	(55)
FePt/TiN/NiFe	interlayer exchange coupling	0.5	*R*_Hall_ vs pulse number	91.17 (three characters)	(11)
W/Pt/Co/NiO/Pt	exchange bias	2.7–3.4	*R*_Hall_ vs pulse number	95.17 (three characters)	this work
				91.25 (four characters)	
				81.25 (Fashion)	

## Supporting Information Available

Eperimental method, details about the GIWAXS pattern, AHE loop under different bias currents, field-free switching loop, switching phase diagram switching percentage, memristive behavior, and several intermediate states of our NiO-based devices, constructed sigmoidal neurons, an ANN for MNIST and Fashion MNIST data sets, effect of LTD and LTP characteristics on recognition accuracy of our proposed NiO-based SOT devices, accuracy of the recognition of three digits and characters, and training accuracy of MNIST and Fashion MNIST image data sets (PDF)
