# Supplementary material for: Neuromorphic Computing with Emerging Antiferromagnetic Ordering in Spin–Orbit Torque Devices
Source: Nano Lett. 2024 Jun 13;24(25):7706–15. doi: 10.1021/acs.nanolett.4c01712 (PMC11212055; doi:10.1021/acs.nanolett.4c01712)
Supplement: Supplementary file 1 — nl4c01712_si_001.pdf [file nl4c01712_si_001.pdf]

Supporting Information

Neuromorphic Computing with Emerging Antiferromagnetic Ordering in Spin-Orbit Torque Devices

Durgesh Kumar Ojha[1,2,3], Yu-Hsin Huang[3], Yu-Lon Lin[3], Ratnamala Chatterjee[2,4], Wen-Yueh Chang[5] and Yuan-Chieh Tseng[1,3]*

[1]International College of Semiconductor Technology, National Yang-Ming Chiao Tung University, Hsinchu 30010, Taiwan, ROC

[2]Magnetics and Advance Ceramics Lab, Department of Physics, Indian Institute of Technology Delhi, Hauz Khas, New Delhi 110016, India.

[3]Department of Materials Science & Engineering, National Yang-Ming Chiao-Tung University, Hsinchu 30010, Taiwan, ROC.

[4]National University of Science and Technology MISiS, Leninskiy Prospect 4,119991 Moscow, Russia.

[5]Powerchip Semiconductor Manufacturing Corporation, Hsinchu 30010, Taiwan, R.O.C

*yctseng1978@nycu.edu.tw

Supporting Sections

S1 Experimental Method

S2 GIWAXS pattern details of NiO-based samples

S3 AHE measurements of our NiO-based SOT devices

S4 Switching loop of NiO-based SOT devices under H_x equal zero

S5 Switching Phase Diagram (SPD) of NiO-based SOT devices

S6 Switching Percentage of NiO-based SOT devices

S7 Memristive behaviour and stable intermediate magnetization states of NiO-based devices

S8 Sigmoidal neuron for NiO-based SOT devices

S9 ANN for MNIST/ Fashion MNIST datasets recognition

S10 Impact of LTD/LTP Response in Neuromorphic Recognition Accuracy

S11 Accuracy of MNIST dataset for recognizing three digits and characters

S12 Training accuracy results of MNIST and Fashion MNIST images

S13 Supporting References

S1 Experimental Method:

In NiO-based sputtered samples, the W, Pt, and Co layers were grown using a DC power of 30 watts under 3 mTorr Ar pressure. The NiO layer was deposited via RF sputtering from the insulating NiO target at 150 watts. The base pressure of the UHV system in each case was 3×10^{-8} Torr. A 3 nm thickness of W was employed as a buffer layer. To see the exchange bias effect in our NiO-based AFM samples, the Vibrating sample magnetometer (VSM) and Physical property measurement system(PPMS) were used. The GIWAXS measurements took place at the 25A beamline of the Taiwan Photon Source at the National Synchrotron Radiation Research Center in Taiwan. The GIWAXS experiments employed an X-ray photon energy of 15 keV. The X-ray incident angle was adjusted to 0.05°, and the exposure time was 3 s. The measurements encompassed a q-range of $0.1-5.3$ Å^{-1}.

NiO-based samples were fabricated by conventional lithography and Ar-ion milling techniques to create a Hall cross-bar with dimensions of 60 μm length and 10 μm width. Electrodes were made using Ti (5nm)/Au (100nm).

The anomalous Hall effect (AHE), Current-induced magnetization switching, multiple hall resistance states, and LTD/LTP response of our NiO-based fabricated hall bar device are assessed by using a Keithley 6221 current source meter and a Keithley 2182A Nanovoltmeter. After the SOT switching loop of a device at H_x = -300 Oe, we applied two pulses with different current amplitudes and signs (+30mA and -30mA for NiO2nm and NiO30nm) under the same H_x = -300 Oe. 25 pulses were chosen, and we observed R_{Hall} vs pulse number response in terms of long-term potentiation (LTP) and long-term depression (LTD) corresponding to positive and negative consecutive pulse currents. This setup is controlled by Labview programming and is fully automatic. The

consecutive pulse current is generated by a Keithley 6221 current source with a pulse width of 500μs and a small reading current of 1 mA.

S2 GIWAXS pattern details of NiO-based samples:

Figures 1 (a), (b), and (c) illustrated in the main context, demonstrate the GIWAXS patterns of NiO-2nm, NiO-15nm, and NiO-30 nm samples, respectively. The exhibited circular bight ring spots correspond to different Bragg diffraction planes, and the experimental observed scattering wave vector magnitude$|\vec{q}|$ obtained from these patterns is 2.83, 3.31,4.66 & 5.42 $Å^{-1}$ for NiO-2nm sample and 2.65, 3.07, 4.31, & 5.04 $Å^{-1}$ for NiO-15nm and NiO-30nm samples respectively. We theoretically calculated the q value for (111), (200), (220), and (311) planes through $|\vec{q}| = 2\pi/d_{hkl}$, (where d_{hkl}, signifies the interplanar spacing of the neighboring (*hkl*)) of NiO-based samples, by considering the lattice constant of Pt and NiO is 3.84 and 4.11 Å under the simple cubic system respectively. This q value is matched with the experimental GIWAXS pattern and the polycrystalline nature of our NiO-based samples is confirmed.

To gain a deeper understanding of the grain orientation-related details, we perform azimuthal integrations at a consistent sweeping angle of 3° while adjusting polar angles Ψ, as illustrated in Figure 1(d) of the main context. The polar angle Ψ= 0° represents the surface normal of the sample and signifies the inclined orientation of the (111) crystallography planes of the NiO films[1]. Figure 1(d) shows the extracted 2-θ profile of NiO-2nm, NiO-15nm, and NiO-30nm samples from their GIWAXS pattern at Ψ= 0° respectively, and it clearly illustrates the Bragg diffraction peaks and the grain orientation of Pt (111) and NiO, (111), (200), (220), and (311) planes for each NiO-based sample respectively.

S3 AHE measurements of our NiO-based SOT devices:

The AHE is a widely recognized occurrence in FM materials. In a uniform FM sample, it exhibits a linear relationship with the OOP magnetization (M_z), represented as $R_{Hall}(H_z) \propto M_z(H_z)$, where R_{Hall} is the Hall resistance[2]. This confirms the H_z^{eff} field, in our proposed NiO-based samples. The AHE loops for NiO-2nm, NiO-15nm, and NiO-30nm devices are presented in Figures S3 (a), (b), and (c), respectively, under different bias currents ranging from +1.0 mA to +21.0 mA (for NiO-2nm), +1.0 mA to +37.0 mA (for NiO-15nm), and +1.0 mA to +29.0 mA (for NiO-30nm), with zero in-plane fields (H_x = 0 Oe). By using these AHE loops, we estimated H_z^{eff} field for each NiO-based device.

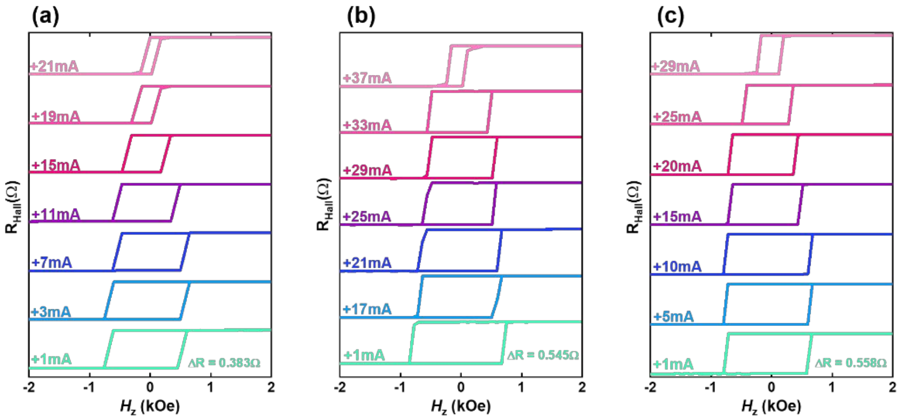

Figure S3: Anomalous Hall effect measurements; (a),(b), and (c) represent the AHE loop of NiO-2nm, NiO-15nm, and NiO-30nm devices respectively, under different bias currents with H_x = 0 Oe.

S4 Switching loop of NiO-based SOT devices under H_x equal zero:

Figure S4 presents the FFS loop of our NiO-based SOT devices, and it can be seen that no complete switching loop is obtained for the NiO-2nm device, which indicates an un-

established Co/NiO coupling which was confirmed by the GIWAXS pattern. In contrast, deterministic FFS switching is observed in 15nm and 30nm NiO devices with switching percentages of ~8% and ~10%, respectively. This was expected due to the perpendicular exchange bias (EB) and effective H_z^{eff} field. In the NiO-15nm device, we noticed a large switching current density compared to 2nm and 30nm, it is because of their large coercive (H_c) field, which may come from weak Co/NiO coupling. Figure S4 illustrates both gradual (NiO-15nm, from up-to-down states) and sharp-linear (NiO-30nm) FFS characteristics, attributed to weak and strong Co/NiO coupling.

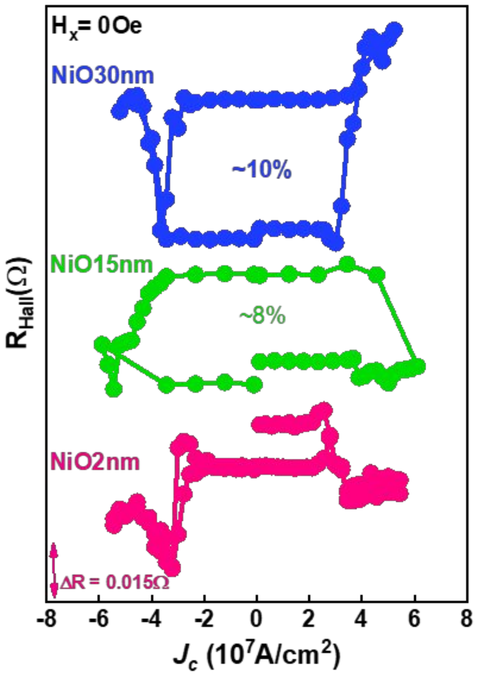

Figure S4: The FFS loop of NiO-based devices: the pink color (bottom) represents an incomplete switching loop corresponding to NiO-2nm, while the deterministic switching loops shown in green (middle) and blue (top) represent NiO-15nm and NiO-30nm devices, respectively.

S5 Switching Phase Diagram (SPD) of NiO-based SOT devices:

The abrupt change in R_{Hall} for a particular current density are indicator of the magnetization switching of the Co layer from up to down (for $J_c \cdot H_x > 0$) and from down

to up (J_c. $H_x < 0$). Figures S5 (a), (b), and (c) demonstrate the variation of critical switching current density (J_c) under different H_x (+3kOe to -3kOe), of NiO-2nm, NiO-15nm, and NiO-30nm devices respectively.

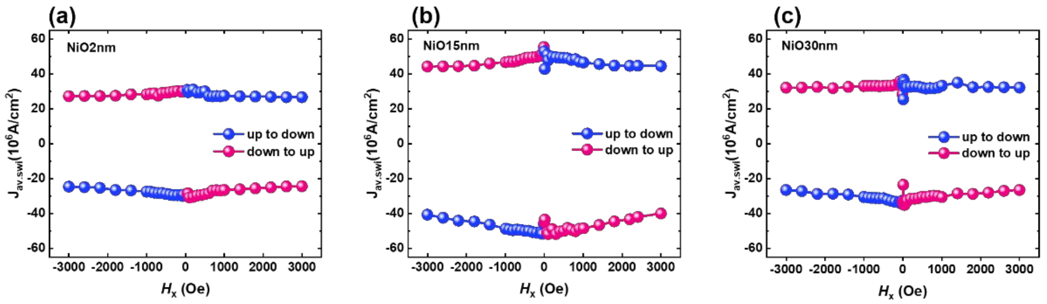

Figure S5: Switching phase diagram of NiO-based SOT devices; (a), (b) and (c) represent the NiO-2nm, NiO-15nm, and NiO-30nm devices respectively. The Blue and pink color balls represent up-to-down and down-to-up switching current variation under different H_x for each device respectively.

From SPD, we find the J_c range for all NiO-based devices under different H_x, which vary from +3kOe to -3kOe, are summarized in the following Table 1.

Critical switching current density J_c (10^6 A/cm^2)	NiO-2nm	NiO-15nm	NiO-30nm
J_c^+	27 to 30	44 to 53	28 to 34
J_c^-	-24 to -29	-40 to -52	-26 to -33

Table 1. The approximate critical switching current range of our NiO-based SOT device under different in-plane bias fields H_x = + 3kOe to -3kOe.

At H_x = 0 Oe, the critical current density (J_c) for the 15nm device is 52.9 × 10^6 A/cm^2 in the down-to-up states and -45× 10^6 A/cm^2 in the up-to-down states. In contrast, for the 30nm device, these values are 33.9 × 10^6 A/cm^2 and -33.3 × 10^6 A/cm^2, respectively.

S6 Switching Percentage of NiO-based SOT devices:

For further confirmation of the perpendicular EB effect in our NiO-based device, we estimated the switching percentage shown in Figure S6, by taking the variation of $\Delta R/\Delta R_{AHE}$, versus H_x, here ΔR is the total change in R_{Hall} under the current induced SOT switching measurements, its positive and negative value corresponding to negative and positive in-plane field direction respectively, and ΔR_{AHE} is the total change in R_{Hall} under AHE measurements, and its value is 0.383Ω, 0.545Ω and 0.558Ω for NiO-2nm, NiO-15nm, and NiO-30nm device at +1.0mA bias current respectively.

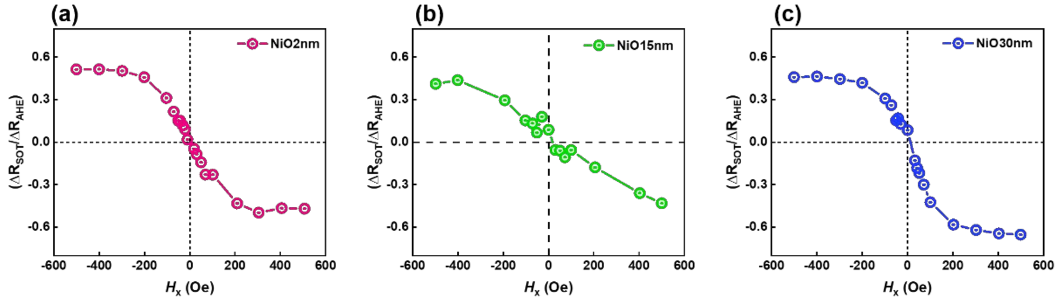

Figure S6: Switching percentage of our NiO-based devices; (a), (b), and (c) illustrate the switching percentage of NiO-2nm, NiO-15nm, and NiO-30nm devices respectively.

It can be seen that the NiO-2nm, graph (Fig.S6(a)) is almost symmetric about H_x = 0 Oe, and no shift along H_x, it supports perpendicular EB and confirms no in-plan EB effect[3]. The minimum switching percentage of ~2% is also confirmed at -10 Oe for NiO-2nm devices. Similarly, NiO-15nm and NiO-30nm (Fig.S6(b), (c)) show no shift along H_x which means the in-plane EB effect is absent in our proposed NiO-based sample. However the asymmetric behavior in terms of switching percentage, which indicates changes in ΔR with the direction of H_x is found in the case of NiO-30nm device. For example, the magnitude of ΔR value for +70 Oe is higher than -70 Oe, we

can see for positive H_x, the switching percentage is higher than negative H_x for the NiO-30nm case, while for the NiO-2nm and 15nm device, the switching percentages is almost same for each ± H_x value. This indicates that there is an effective field along the z direction which supports Co layer magnetization switching and enhanced ΔR for the positive direction of H_x in the case of thick NiO. The approximate switching percentage ratio of our NiO-based devices under different H_x is summarized in Table 2. The enhanced switching percentage of the NiO-30nm device compared to NiO-2nm and NiO-15nm is attributed to the robust perpendicular EB effect in the NiO-30nm device and confirms that the observed FFS in our device is due to the perpendicular EB, not due to in-plane EB effect.

H_x (Oe)	NiO-2nm (%)	NiO-15nm (%)	NiO-30nm(%)
0	1.5	8	10
+50	14	6	22
-50	15	7	16
+500	47	43	65
-500	51	41	46

Table 2. The approximate switching percentage ratio of our NiO-based SOT device under H_x = 0 Oe, ±50 Oe, and ±500 Oe.

S7 Memristive behaviours and stable intermediate magnetization states of NiO-based devices:

We examined the memristive behavior of our NiO-based devices by sweeping pulse current under H_x= -300 Oe, as shown in Figure S7. Figures S7(a), (b), and (c) illustrate the memristive behaviors of NiO-2nm, NiO-15nm, and NiO-30nm devices respectively. The change in R_{Hall}, which indicates the switching ratio, increases with higher maximum pulse current amplitudes (20mA to 45 mA for NiO-2nm, 40mA to 54 mA for NiO-15nm, and 29mA to 45mA for NiO-30nm devices). This suggests that different current densities in our NiO-based hall bar device could modulate the intermediate magnetization states in terms of R_{Hall}. A significant characteristic of memristors is their

capability to retain multiple intermediate R_{Hall} levels instead of binary states in memory[4]. We observed these multiple intermediate R_{Hall} states in our NiO-based device, illustrated in Figure S7 (d)-(f). Figure S7 (d), (e), and (f) correspond to NiO-2nm, NiO-15nm, and NiO-30nm hall bar devices respectively. To achieve these intermediate magnetization states in terms of R_{Hall}, we selected varying pulse current amplitudes (-22 mA, -25 mA, -28 mA, and -32 mA for NiO-2nm) and initialized pulse currents (40 mA for NiO-2nm) for each device as depicted in their respective figures S7(d)-(f). The selection of different pulse current amplitudes and reversal-sign initialized currents to attain the multiple R_{Hall} states of devices depends on the switching loop at different maximum current amplitudes (as shown in Figure S7(a), (b), and (c)) for each respective device, reflecting their memristive behavior.

The NiO-2nm and NiO-15nm devices displayed four stable intermediate magnetization states (R_{Hall}), whereas the NiO-30nm device showed three stable states. This suggests that NiO-2nm and NiO-15nm function as more effective mersisters compared to NiO-30nm, indicating that strong Co/NiO exchange coupling suppresses memristivity in the NiO-30nm device.

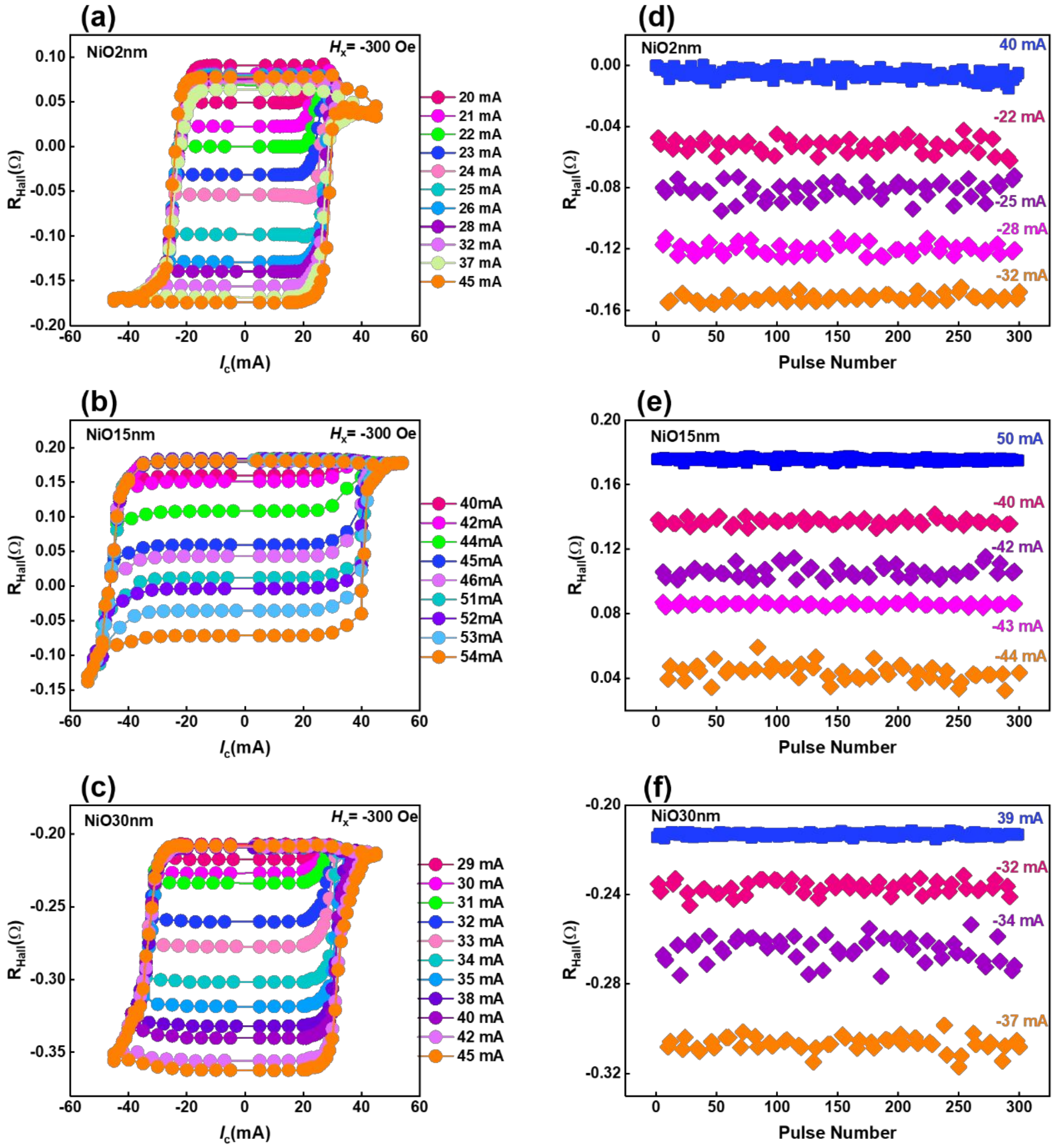

Figure S7: Memristive behavior and intermediate magnetization states (R_{Hall}) of our NiO-based SOT devices. Figures (a), (b), and (c) illustrate the memristive characteristics of NiO-2nm, NiO-15nm, and NiO-30nm hall bar devices, respectively, under H_x=-300Oe. Different pulse current amplitudes (20 mA to 45 mA for NiO-2nm, 40 mA to 54 mA for NiO-15nm, and 29 mA to 45 mA for NiO-30nm) indicate the maximum pulse current amplitude during the switching loop. Figures (d), (e), and (f) depict the stable intermediate magnetization states of these devices in terms of R_{Hall}.

S8 Sigmoidal neuron for NiO-based SOT devices:

The basis for the sigmoidal neuron in our NiO-based device is the gradual multiple intermediate magnetization state, which serves as the sigmoid activation function (shown in Figure 5(a)-(b) in the manuscript). Jing Zhou et.al utilized an $L1_1$-CuPt/CoPt bilayer as a sigmoidal neuron for neuromorphic computing[5], while Kaifeng Dong et al. used $L1_0$ FePt for enhanced MNIST character recognition accuracy[6]. The sigmoid activation function for each NiO-based device is derived from its respective experimental SOT switching loop, illustrated in Figure 5 (a) of the manuscript. We extracted multiple intermediate magnetization states from the experimental SOT switching loop of the NiO-based device, represented as R_{Hall} data points in the shaded region of Figure 5(a) in the manuscript. These states were then fitted with the mathematical sigmoid function expressed as $\boldsymbol{y = A_2 + \frac{A_1 - A_2}{1 + e^{(x - x_0)/dx}}}$.

We obtained the fitted sigmoid function parameters (A_1, A_2, x_o, dx) and utilized these in Python code as the activation function for the hidden layer in the neural network during MNIST pattern recognition accuracy for the NiO-2nm device, as shown in the inset of Figure S8-(a). This procedure was repeated for each NiO-based device to extract its respective sigmoid function as the activation function, which was then utilized to simulate the MNIST dataset recognition accuracy for each device.

For the NiO-2nm, 15nm, and 30nm devices, we repeated the SOT switching loop measurements under the same conditions and constructed approximately the same sigmoid activation functions (Fig. S8(b)-(d)). The SOT switching loop was iterated twice for the NiO-2nm device and three times for the NiO-15nm and 30nm devices respectively, under H_x = -300 Oe. The corresponding sigmoid function was obtained for all NiO-based devices, as shown in Figure S8. Figures S8 (b), (c), and (d) demonstrate the constructed sigmoidal activation function of NiO-2nm, 15nm, and 30nm SOT devices respectively. The optimal sigmoid function of all devices was

chosen in this process for the MNIST pattern recognition task and is illustrated in the primary context of Figure 5(b).

The fitted sigmoid activation function corresponding to NiO-2nm is better than other NiO devices. It indicates that the extracted sigmoidal activation function is influenced by Co/NiO coupling. Optimal performance occurs with low coupling, producing the most effective sigmoid function. However, strong coupling adversely affects the function, leading to reduced recognition accuracy.

Spintronic devices possess essential features like nonlinearity, stochasticity, and nonvolatility, making them indispensable for neuromorphic computing. The sigmoid activation function introduces crucial nonlinearity into neural networks, enabling them to handle tasks such as complex pattern recognition, classification, and regression. Its smoothness aids in stable and efficient learning, especially with gradient descent-based optimization like backpropagation. Additionally, the sigmoid function's ease of implementation and versatility in adjustment make it widely used in traditional artificial neural networks (ANN)[5,6].

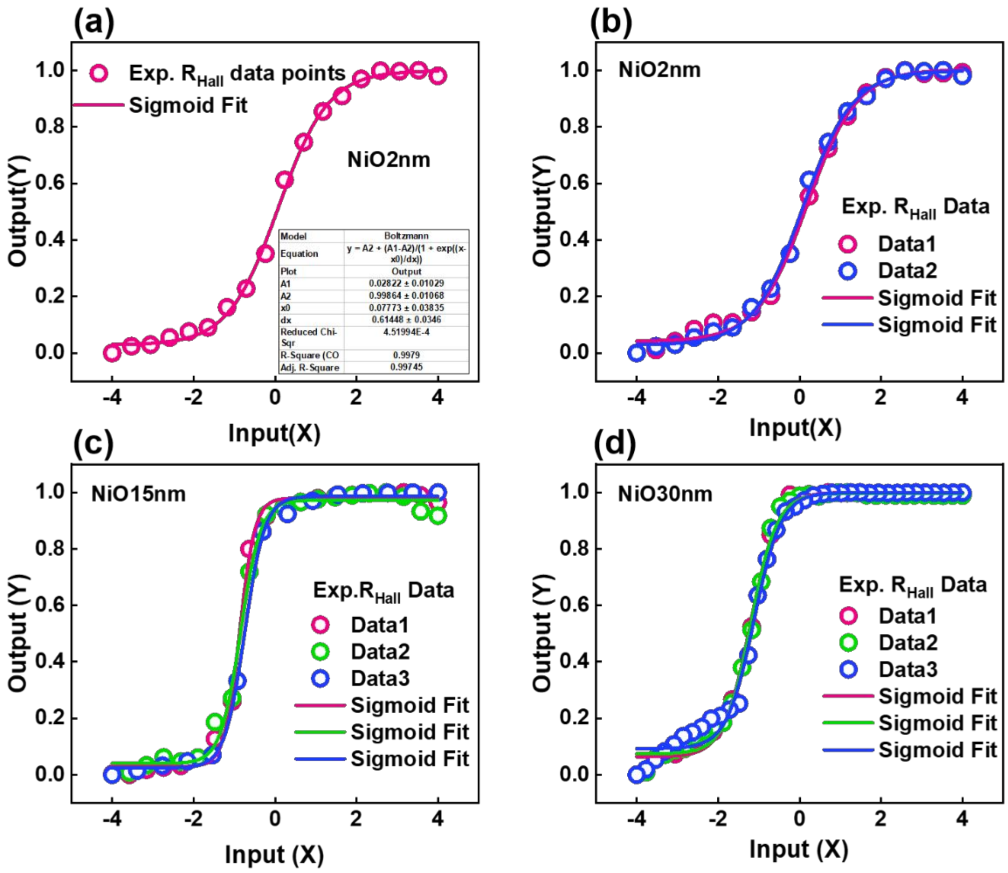

Figure S8: Sigmoidal activation function derived from experimental SOT switching loop; (a) Sigmoid activation function of NiO2nm device. The pink circle represents the R_{Hall} intermediate multistate of the NiO-2nm SOT switching loop as shown in the manuscript Figure 5(a). The pink line shows the fitted sigmoid function, and the inset box illustrates the fitted parameters, which were used in our Python code for NiO2nm neuromorphic recognition performance. (b), (c), and (d) represent the exp. R_{Hall} data point with respective fitted sigmoid functions of NiO2nm, NiO15nm, and NiO30nm respectively under repeated SOT switching loop. The different circles of each NiO-based device represent the repeated intermediate magnetization states (Exp. Data) in terms of the R_{Hall} of each device.

S9 ANN for MNIST/ Fashion MNIST datasets recognition:

The implemented ANN in Python code for MNIST and Fashion MNIST pattern recognition utilizes a simple 3-layer MLP neural network. It consists of an input layer with 784 neurons, reflecting the 28×28 pixel values of each MNIST image transformed into a 1-dimensional array. For the MNIST datasets, there are two hidden layers with 10 neurons each, while for Fashion MNIST images, there are two hidden layers with 50 neurons each. The output layer varies with 4 neurons for MNIST (digits and characters) and 2 neurons for Fashion MNIST, corresponding to the number of recognition image patterns.

Each neuron in the hidden layers performs a weighted sum of its inputs from the previous layer and applies a fitted sigmoid activation function, as illustrated in Figure 5(b) of the manuscript. The sigmoid function introduces non-linearity into the network, enabling it to learn complex patterns and relationships in the data.

During training, the ANN utilizes the R_{Hall} vs pulse number states (150 R_{Hall} for MNIST and 300 R_{Hall} for Fashion MNIST) of our NiO-based device, depicted in Figure 4(d)-(f) in the main context, as synaptic weights between the input and output layers. The training process employs traditional gradient descent and backward propagation algorithms[6,7], with a mean square error loss function. Different learning rates are applied for MNIST (0.1) and Fashion MNIST (0.01) to improve MNIST pattern recognition accuracy. The output layer employs the SOFTMAX function for classification, generating a four-class output for MNIST and a two-class output for Fashion MNIST datasets. The predicted label class is determined by selecting the output neuron with the highest likelihood. The architecture of the ANN used for MNIST dataset recognition is depicted in Figure S9.

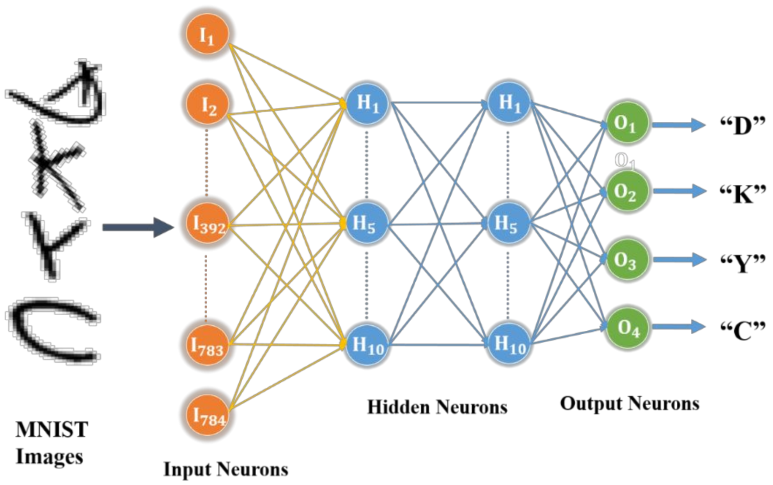

Figure S9: The implemented ANN for MNIST image recognition accuracy consists of three layers with 784 input neurons, two hidden layers with 10 neurons each, and an output layer with 4 neurons. Each MNIST image character is 28×28 pixels in size.

S10 Impact of LTD/LTP Response in Neuromorphic Recognition Accuracy:

The low-nonlinear and symmetric LTD/LTP response under pulse currents is advantageous for high recognition accuracy in ANN[8-11]. The NiO-2nm device shows a low nonlinear, symmetric, gradual response of LTD and LTP with increasing pulse number (Fig.S10(a)). NiO-15nm devices exhibit almost linear LTP and slightly higher nonlinear LTD compared to the 2nm device (Fig.S10(b)). Conversely, the NiO-30nm device demonstrates a high nonlinear LTP behaviour compared to 2nm and 15nm NiO devices, with sharp changes in LTD and LTP (Fig.S10(c)). This nonlinearity and asymmetry in the LTD and LTP behaviour of NiO-30nm devices adversely affects their prediction accuracy[11]. Additional cases confirm that the NiO-2nm and 15nm device's superiority is associated with its distinctive synaptic behavior and the optimal extraction of a sigmoidal activation function. For this confirmation, we examined two more cases, in the first case, the fitted sigmoid function displayed in Figure 5(b) of the main context is utilized as an activation function of the hidden layer, and weight updating is achieved using an ideal synapse, referred to as “case1” second, the ideal

sigmoid function as the activation function, and the synaptic weight updated by 150 R_{Hall} states, referred as to “case2”. The simulation results demonstrate that "Case 1" outperforms "Case 2," confirming the superiority of NiO-2nm and 15nm devices over NiO-30nm, as illustrated in Figure S10 (d) and (e) respectively. The accuracy results of the simulation tests on NiO-2nm, NiO-15nm, and NiO-30nm neuromorphic devices are summarized in Table 3. Case 1 shows a slight difference for all devices, which indicates a low effect of the sigmoid activation function on recognition accuracy, while Case 2 reveals a significant disparity, suggesting that the lower accuracies in the NiO-30nm device are attributed to its pronounced nonlinear LTD/LTP response with pulse number. The NiO-15nm device exhibited slightly higher accuracy (~ 1%) than the NiO-2nm device in case 2, attributed to the more linear LTD response of NiO-15nm compared to NiO-2nm. The simulation results confirm that the superior recognition accuracy of the NiO-2nm and NiO-15nm devices over NiO-30nm is primarily influenced by experimentally extracted high nonlinear synaptic weight, possibly due to strong Co/NiO coupling. This finding aligns with the conclusion drawn from Figure S7, which indicates that NiO-2nm and NiO-15nm devices exhibit better memristive properties than NiO-30nm devices.

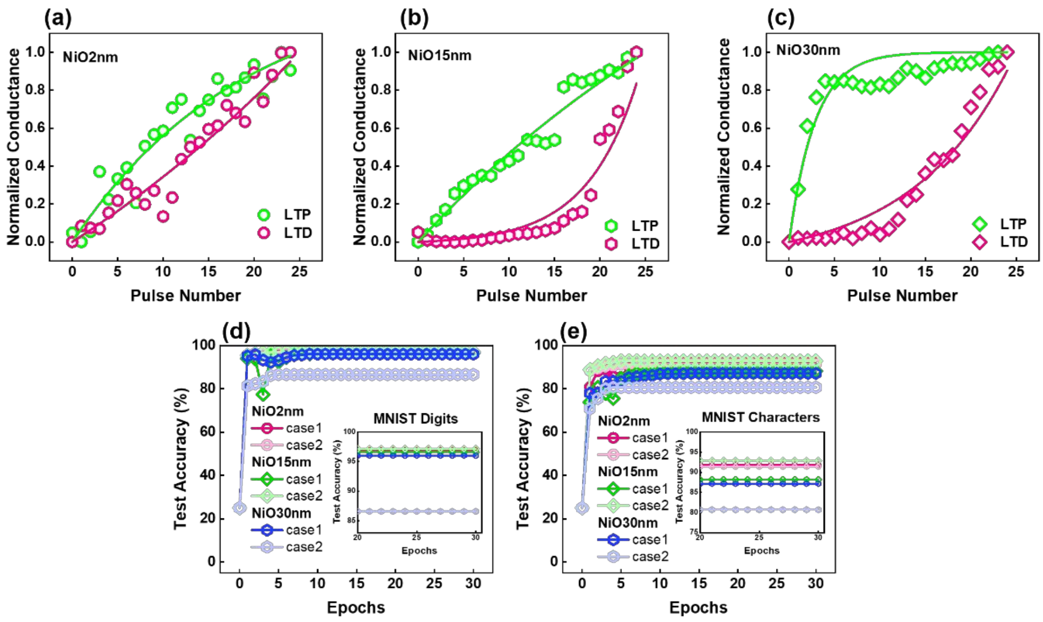

Figure S10: Effect of nonlinearity LTD/LTP characteristics on recognition accuracy of MNIST datasets. (a), (b), and (c), demonstrate the normalized conductance versus pulse number response under non-linearity analysis of NiO-2nm, NiO-15nm, and NiO-30nm devices respectively. (d) MNIST digits and (e) MNIST characters recognition accuracy of our NiO-based neuromorphic devices under case 1 and case 2 respectively. The pink, green, and blue colors illustrate the recognition accuracy of the NiO-2nm,15nm, and 30nm devices respectively.

MNIST Datasets	Cases	NiO-2nm device test accuracy(%)	NiO-15nm device test accuracy(%)	NiO-30nm device test accuracy(%)	Synaptic weight
Handwritten	Case1	96.96	96.59	96.07	150 R_{Hall}
Digits[1,2,4,6]	Case2	96.66	97.17	86.61	
Handwritten	Case1	91.91	88.25	87.16	150 R_{Hall}
Characters[d,k,y,c]	Case2	91.44	92.87	80.75	

Table 3: MNIST images pattern recognition test accuracy of NiO-based neuromorphic devices under case1 (exp. sigmoidal neuron and ideal synapse) and case2 (ideal sigmoid neuron and exp. constructed synaptic weight).

S11 Accuracy of MNIST dataset for recognizing three digits and characters:

We used three digits “2”, “4” and “6” and three characters “d”, “e”, and “p” to test our device accuracy performance. The ANN consists of an input layer with 784 neurons, two hidden layers, each comprising 3 neurons, and an output layer with 3 neurons. 17,718 MNIST digits and 14,400 MNIST characters were utilized to train our devices and their accuracy is shown in Fig. S11(b), and (d) respectively. Subsequently, 2972

images of digits and 2400 images of characters are tested, and their best accuracy is shown in Figure S11 (a) and (c) respectively. For the NiO-2nm, 15nm, and 30nm devices, we achieved ~ 95.4%, ~92.1%, and ~91% test accuracy in the case of MNIST digits respectively, which is close to their ideal accuracy of 97.31%. We used the same respective synaptic weights of 150 R_{Hall} and sigmoidal neurons for all NiO-based neuromorphic devices. The simulation results reveal the higher test accuracy of the NiO-2nm device under exp. synapse and neuron are ~ 95% for MNIST characters, which is close to their ideal accuracy of 96.63% respectively. In contrast, NiO-15nm and 30nm devices yielded lower accuracy for MNIST characters cases.

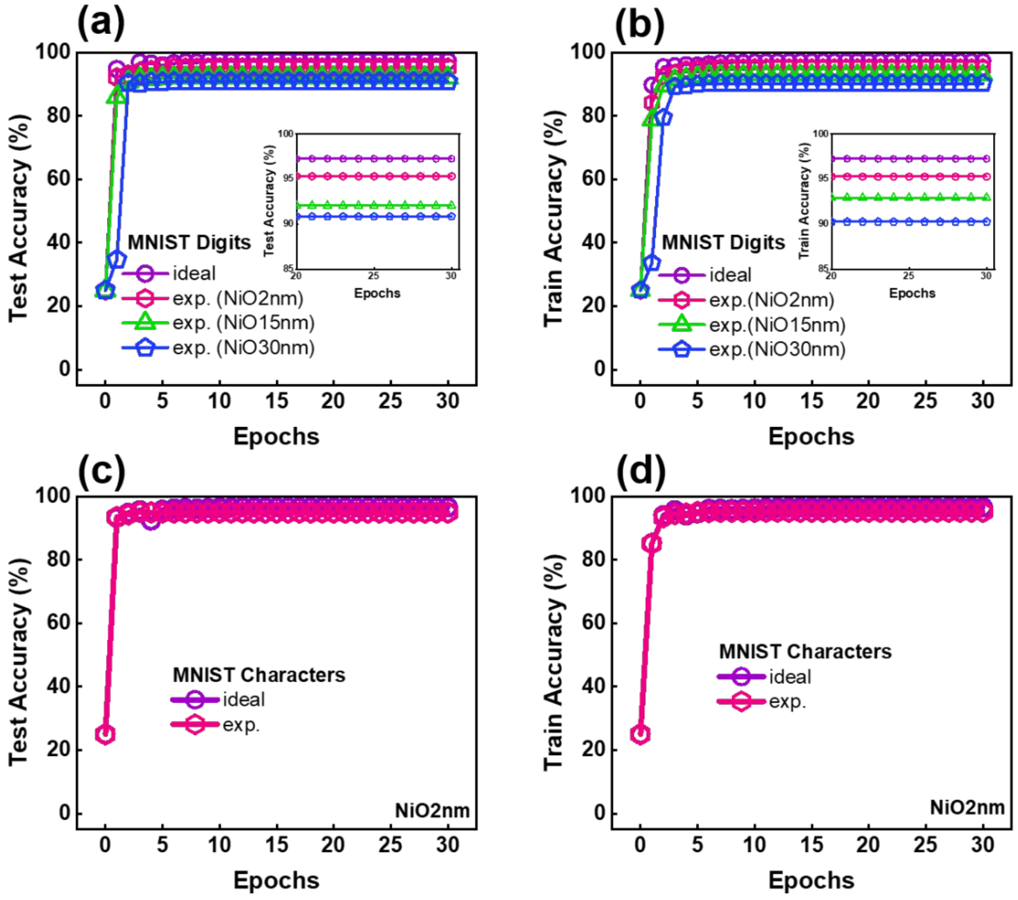

Figure S11: Recognition accuracy of three MNIST digits and characters; (a) and (c) illustrate the MNIST digits and characters recognition accuracy performance of our NiO-based devices respectively. (b) and (d) present the corresponding training accuracy

of the devices. The purple circle represents the ideal synapse and neuron case, the pink, green, and blue colors correspond to the NiO-2nm,15nm, and 30nm respectively.

S12 Training accuracy results of MNIST and Fashion MNIST images:

In the case of the Fashion MNIST dataset, 300 R_{Hall} states, depicted in Fig. S12(a)-(c), served as synaptic weights. The experimental sigmoidal neurons used for all respective NiO devices are shown in Figure 5(b) in the main text.

We trained 24460 images of "1", "2", "4", and "6" MNIST handwritten digits and 19200 images of "d", "k", "y", and "c" characters through ANN, implemented by an artificial synapse (150 R_{Hall} vs pulse number states) and sigmoidal neuron of our NiO-based SOT devices. The training accuracy is shown in the following Figure S12 (d). Similarly, 12000 Fashion MNIST images of pullovers and coats are used for training purposes, and their accuracy is illustrated by Figure S12 (e).

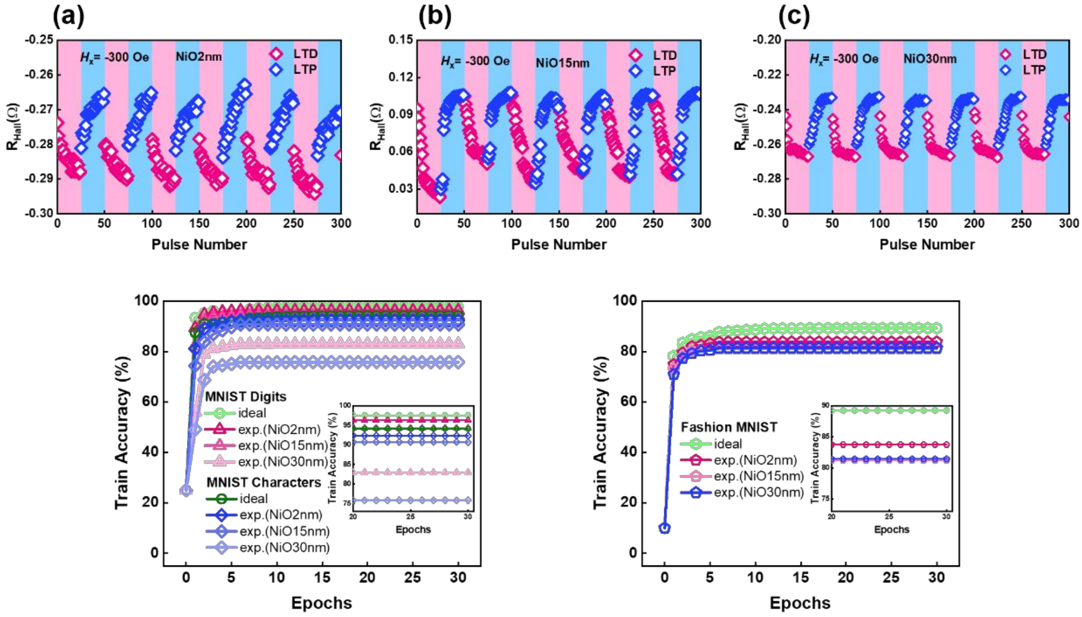

Figure S12: MNIST and Fashion MNIST images train accuracy of NiO-based device; (a), (b), and (c) represent the 300 R_{Hall} response under pulse current of NiO-2nm, NiO-15nm, and NiO-30nm devices respectively. (d) MNIST digits and MNIST characters are trained from our NiO–based devices and their respective train accuracy is illustrated.

The light and dark green circles represent the ideal case, for digits and characters respectively. The dark and light pinks represent the exp. case of NiO-2nm, NiO-15nm, and NiO-30nm devices for MNIST digits accuracy. Similarly, the dark and light blues correspond to NiO-2nm, NiO-15nm, and NiO-30nm characters accuracy respectively. (e) Fashion MNIST training accuracy of our proposed NiO-based SOT devices demonstrated. The light green color circle represents the ideal case while the pink, light pink, and blue represent the exp. case accuracy of NiO-2nm, 15nm, and 30nm devices' respectively.

S13 Supporting References

[1] Hsin, T.C.; Lin, H.Y.; Lin, Y.L.; Chen, J.W. and Tseng, Y.C. Resistive Memristor Coupled with Multilevel Perpendicular Magnetic States. *ACS Appl. Electron. Mater.* **2023**, 5 (11), 6315−6323.

[2] Tai, L.; Dai, B.; Li, J.; Huang, H.; Chong, S.K.; Wong, K.L.; Zhang, H.; Zhang, P.; Deng, P.; Eckberg, C.; Qiu, G. Distinguishing the Two-Component Anomalous Hall Effect from the Topological Hall Effect. *ACS Nano* **2022**, 16 (10), 17336-17346.

[3] Razavi, S.A.; Wu, D.; Yu, G.; Lau, Y.C.; Wong, K.L.; Zhu, W.; He, C.; Zhang, Z.; Coey, J.M.D.; Stamenov, P.; Amiri, P.K. Joule heating effect on field-free magnetization switching by spin-orbit torque in exchange-biased systems. *Phys. Rev. Appl.* **2017**, 7 (2), 024023.

[4] Tao, Y.; Sun, C.; Li, W.; Wang, C.; Jin, F.; Zhang, Y.; Guo, Z.; Zheng, Y.; Wang, X.; Dong, K. Spin-Orbit Torque-Driven Memristor in $L1_0$ FePt Systems with Nanoscale-Thick Layers for Neuromorphic Computing. *ACS Appl. Nano. Mater.* **2023**, 6 (2), 875-884.

[5] Zhou, J.; Zhao, T.; Shu, X.; Liu, L.; Lin, W.; Chen, S.; Shi, S.; Yan, X.; Liu, X.; Chen, J. Spin-Orbit Torque-Induced Domain Nucleation for Neuromorphic Computing. *Adv. Mater.* **2021**, 33 (36), 2103672.

[6] Dong, K.; Guo, Z.; Jiao, Y.; Li, R.; Sun, C.; Tao, Y.; Zhang, S.; Hong, J You, L. Field-Free Current-Induced Switching of $L1_0$-Fe Pt Using Interlayer Exchange Coupling for Neuromorphic Computing. *Phys. Rev. Appl.* **2023**, 19 (2), 024034.

[7] Li, R.; Song, M.; Guo, Z.; Li, S.; Duan, W.; Zhang, S.; Tian, Y.; Chen, Z.; Bao, Y.; Cui, J.; Xu, Y. In-Memory Mathematical Operations with Spin-Orbit Torque Devices. *Adv. Sci.* **2022**, 9 (2), 2202478

[8] Choi, S.; Yang, J.; Wang, G. Emerging memristive artificial synapses and neurons for energy-efficient neuromorphic computing. *Adv. Mater.* **2020**, *32* (51), 2004659.

[9] Kaushik, D.; Singh, U.; Sahu, U.; Sreedevi, I.; Bhowmik, D. Comparing domain wall synapse with other non-volatile memory devices for on-chip learning in analog hardware neural network. *AIP Adv.* **2020**, *10* (2), 025111.

[10] Fu, J.; Liao, Z.; Wang, J. Memristor-based neuromorphic hardware improvement for privacy-preserving ANN. *IEEE Trans. Very Large Scale Integr. VLSI Syst.* **2019**, 27 (12), 2745-2754.

[11] Sun, X.; Yu, S. Impact of non-ideal characteristics of resistive synaptic devices on implementing convolutional neural networks. *IEEE J. Emerg. Sel. Top. Circuits* 2019, *9* (3), 570-579.
